# Statistical Analysis on the Mechanical Properties of Magnesium Alloys

**DOI:** 10.3390/ma10111271

**Published:** 2017-11-06

**Authors:** Shengfeng Guo, Ruoyu Liu, Xianquan Jiang, Hongju Zhang, Dingfei Zhang, Jingfeng Wang, Fusheng Pan

**Affiliations:** 1 Faculty of Materials and Energy, Southwest University, Chongqing 400715, China; lryzczy@163.com (R.L.); jsq89@swu.edu.cn (X.J.); 2 National Engineering Research Center for Magnesium Alloys, Chongqing University, Chongqing 400044, China; zhanghongju@cqu.edu.cn (H.Z.); zhangdingfei@cqu.edu.cn (D.Z.); jfwang@cqu.edu.cn (J.W.); fspan@cqu.edu.cn (F.P.)

**Keywords:** magnesium alloys, mechanical properties, Weibull statistics

## Abstract

Knowledge of statistical characteristics of mechanical properties is very important for the practical application of structural materials. Unfortunately, the scatter characteristics of magnesium alloys for mechanical performance remain poorly understood until now. In this study, the mechanical reliability of magnesium alloys is systematically estimated using Weibull statistical analysis. Interestingly, the Weibull modulus, m, of strength for magnesium alloys is as high as that for aluminum and steels, confirming the very high reliability of magnesium alloys. The high predictability in the tensile strength of magnesium alloys represents the capability of preventing catastrophic premature failure during service, which is essential for safety and reliability assessment.

## 1. Introduction

Magnesium alloys are well known for their lightweight, high specific strength, high recycling ability, and excellent damping capacity [1,2,3,4]. Therefore, the use of magnesium alloys has been significantly increasing worldwide over the past years. Unfortunately, the macroscopic brittle fracture of most magnesium alloys, through cleavage and quasi-cleavage fracture modes [5], may raise a concern regarding the stability of characteristic strength, as extensively revealed in Mg-based glassy alloys [6]. Such a deleteriously flawed sensitivity behavior of magnesium alloys potentially presents a severe problem for their application, which has unfortunately received no attention to date.

There are many approaches to quantify the effect of statistically distributed flaws or defects on the mechanical properties of a brittle material. Among these approaches, Weibull statistical analysis has been widely used to describe the reproducibility of fracture strength [7]. For example, numerous Weibull models have been historically applied to rationalize the strength scatter in many ceramics and brittle metals [8,9]. Nevertheless, extremely rare data are available on the Weibull statistical distribution of strength for magnesium alloys. In this work, we employ Weibull statistical analysis to evaluate the strength reliability of magnesium alloys; the statistical distribution of strength is found to be highly correlated with the material’s specific microstructural state or feature. Our finding provides insight into understanding and stabilizing magnesium alloys by tailoring processing protocols.

## 2. Experimental section

Mg-6Zn-1Mn (ZM61) was prepared by vacuum induction melting under an Ar atmosphere using commercial high purity Mg (99.9%), Zn (99.9%) and Mg-4%Mn master alloys (ZG-0.01, Jinzhou, China). The alloy rods (80 mm in diameter and 250 mm in height) were machined and homogenized at 330 °C for 24 h. Then, they were immediately inserted into the extrusion chamber (350 °C) and extruded to bars with a diameter of 16 mm under an extrusion ratio of 25. Subsequently, the extruded bars were solution treated at 420 °C for 2 h followed by water quenching and then artificially aged at 180 °C for 16 h. The detailed processing of the extruded and aged ZM61 magnesium alloys can be seen elsewhere [10]. The microstructure of the extruded and aged alloys was examined by scanning electron microscopy (SEM, TESCAN VEGA 3 LMH, Brno, Czech Republic) and a transmission electron microscope (TEM, ZEISS LIBRA 200FE, Heidenheim, Germany), respectively. The uniaxial tensile characteristics of dogbone-like specimens (50 mm in gauge length and 5 mm in gauge diameter) were determined on a universal testing machine with a strain rate of 2 mm/min. The fracture morphologies were also examined with an SEM (JEOL JSM-7800F, Tokyo, Japan).

## 3. Results and Discussion

**Figure 1:**
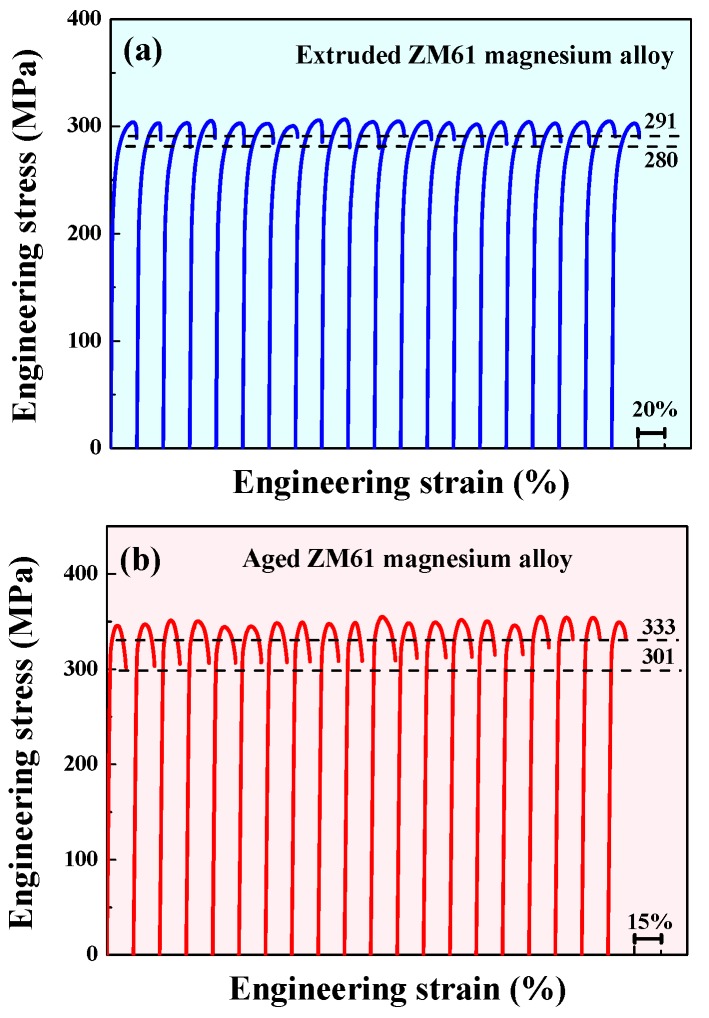
Tensile stress-strain curves of the ZM61 magnesium alloys: (**a**) extruded and (**b**) aged.

Twenty samples were tested for each alloy, and all the stress-strain curves are shown in Figure 1a,b for extruded and aged ZM61 magnesium alloys, respectively. The apparent strength for each experiment was surprisingly uniform, especially for the extruded alloys. The 0.2% offset yield strength (*σ_y_*) for the extruded alloy ranged from 206 to 212 MPa, with a variation of only ±0.7% around its mean value. The ultimate tensile strength (*σ_u_*) and fracture strength (*σ_f_*) ranged from about 300 to 307 MPa and 280 to 291 MPa, with a variation of ±0.5% and ±1.3% around their corresponding mean values, respectively. The statistical result for the extruded alloys demonstrated a very narrow distribution range for the *σ_y_*, *σ_u_* and *σ_f_*. For the aged alloys, the *σ_y_* ranged from 307 to 320 MPa, with a variation of ±1.3% around its average value. The *σ_u_* and *σ_f_* ranged from about 345 to 355 MPa and 302 to 333 MPa, with a variation of ±0.9% and ±3% around their corresponding average values, respectively. Interestingly, the strength of all the magnesium alloys matched the statistical trend and displayed a narrow distribution, which was evidently very different from common brittle materials, the tensile strength of which is usually highly scattered [8].

Weibull statistics is a well-established characterization tool in the field of fracture strength of brittle materials. Weibull related the cumulative failure probability $P_{f}$ of volume V of a material under a uniaxial tensile stress *σ* using the following relationship [8]:

(1) $$P_{f}=1-\exp\left[-V{\left(\frac{\sigma -\sigma_{u}}{\sigma_{0}}\right)}^{m}\right]$$

where *σ*_0_ is a scaling parameter, *m* is the Weibull modulus and V is a normalized volume of the tested sample. The *σ_u_* is the location parameter, denoting the stress at which there is a zero failure probability; it is usually taken as zero for the safest assumption [11]. For *N* nominally identical specimens ranked from the weakest (*i* = 1) to the strongest (*i* = *N*), the failure probability $P_{f}$ of the *i*th one is calculated using the following equation [12]:

(2) $$P_{f,i}=\frac{n_{i}-0.5}{N}$$

where $n_{i}$ is the *i*th sample ($n_{i}$ = 1, …, *N* experiments) and *N* is the total number of samples tested. These results were then plotted in the usual double logarithmic form of the Weibull expression. Therefore, the parameters of the Weibull distribution could be obtained by linearizing Equation (1).

(3) $$\ln\left[\ln\left(\frac{1}{1-P_{f}}\right)\right]=\ln V+m\ln\sigma -m\ln\sigma_{0}$$

By fitting a straight line to $\ln\left[\ln\left(1/1-P_{f}\right)\right]$ as a function of lnσ, the Weibull modulus *m* was simply the slope, and the scaling parameter *σ*_0_ could be determined from the intercept. The coefficient of determination $R_{}^{2}$ has been commonly used as a measure of the goodness of fit. The higher the value of $R_{}^{2}$, the more likely the data are to follow the distribution being tested. Recently, Tiryakioglu et al. [13] ran Monte Carlo simulations to determine the critical points of $R_{}^{2}$ and proposed that the following formula can be used to evaluate the goodness-of-fit tests for sample sizes between 5 and 100:

(4) $$R_{0.05}^{2}=1.0637-\frac{0.4174}{n^{0.3}}$$

If the calculated $R_{}^{2}$ is higher than $R_{0.05}^{2}$, then it can be concluded that the data indeed came from a Weibull distribution. The Weibull fit was acceptable. On the contrary, the two-parameter Weibull analysis was not valid if $R_{}^{2}$ < $R_{0.05}^{2}$.

**Figure 2:**
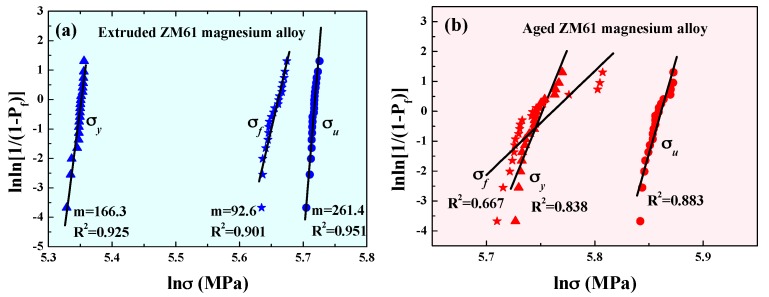
Weibull plot of tensile strength of the ZM61 magnesium alloys: (**a**) extruded and (**b**) aged.

Figure 2 shows the Weibull plots in the form suggested by Equation (3) for the extruded (a) and aged (b) magnesium alloys. From Figure 2a, a very good linear relationship of the *σ_y_*, *σ_u_* and *σ_f_* was observed. The coefficients of determination, *R*^2^, for the *σ_y_*, *σ_u_* and *σ_f_* were 0.925, 0.951 and 0.901, respectively, which were all higher than the value of $R_{0.05}^{2}$ (0.894), suggesting that the experimental data could be reasonably described by the Weibull distribution equation. Linear least squares fitting of Equation (3) was performed for these data given the Weibull modulus *m* of the *σ_y_*, *σ_u_* and *σ_f_* as 166.3, 261.4 and 92.6 for the extruded alloys, respectively. Surprisingly, the failure Weibull modulus *m*, which ranged from 90 to 100, was as high as the ductile aluminum and steel [8]. Since the *m* value reflected the degree of variation in the strength of the samples tested, a higher *m* value implied a narrower distribution of fracture stresses and a higher reliability. Typical values of the Weibull modulus *m* for some materials including Mg-based bulk metallic glasses [14] were summarized and are listed in Table 1. Although magnesium alloys are often regarded as macroscopically brittle materials, it was interesting to observe a very high reliability from the rather uniform strength data and large Weibull modulus. Furthermore, as shown in Figure 2b, the Weibull plots of the *σ_y_*, *σ_u_* and *σ_f_* for the aged alloys displayed a relatively poor linear relationship, especially for *σ_f_*. The coefficients of determination, R^2^, from the linear least squares fitting method were 0.838, 0.883, 0.677 for the *σ_y_*, *σ_u_* and *σ_f_*, respectively. The $R_{}^{2}$ was much lower than the value of $R_{0.05}^{2}$ (0.894), implying that the experimental data for aged alloys were not acceptable based on the two-parameter Weibull distribution [15]. We also noticed significant deviation of some points from the fitting line at high values of ln(*σ_f_*). This was very different from the previous report where the assumption of the threshold was zero, which was no longer appropriate for the two-parameter distribution. This is still an open question and implies that the Weibull analysis may be upgraded to a three-parameter version for fitting. When considering the location parameter *σ_u_*, the three-parameter Weibull model has also been suggested as a more interpretable and accurate reliability assessment [16].

**Figure 3:**
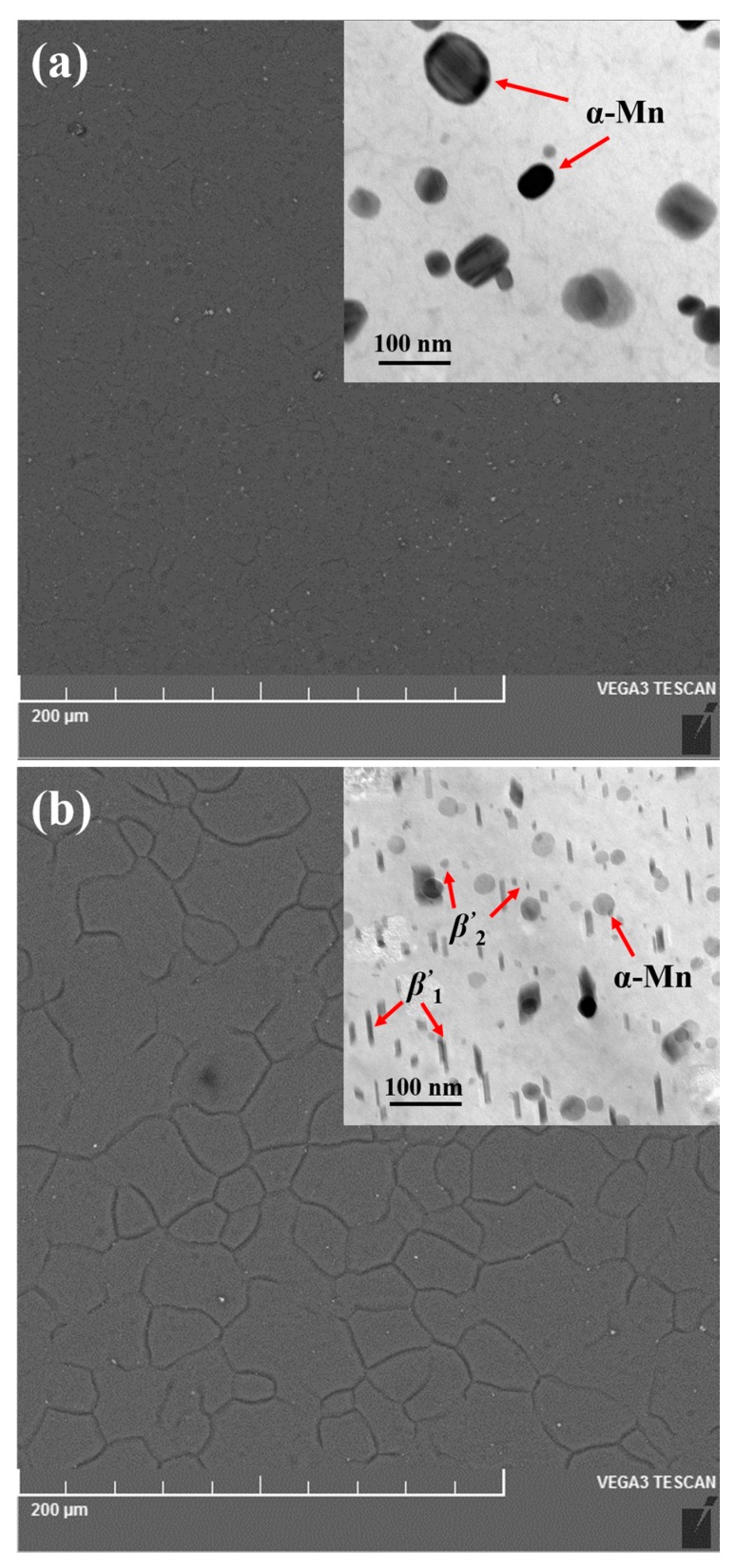
The SEM images and the bright-field TEM images of the microstructure for the ZM61 magnesium alloys: (**a**) extruded and (**b**) aged.

The tensile failure strength *σ_f_* was very sensitive to interior flaws and cracks. For the extruded magnesium alloys, flaws such as shrinkage porosity, oxidation slags and cracks in the as-cast stage were basically eliminated during the hot extrusion [17]. Figure 3a shows the backscattered electron imaging in the SEM of the extruded alloy; after a complete solid solution heat treatment, only a few of the *α*-Mn phases were precipitated for the extruded alloy, which could be further confirmed by the bright field mode of the TEM as shown in the inset. Due to the small amount of precipitation with sparse distribution, it has little effect on the variation of mechanical properties. After the hot extrusion process, the microstructure of the alloy shows a relatively uniform and fine structure. Therefore, a uniform failure strength and large Weibull moduli *m* were attained. However, for the aged alloy as shown in Figure 3b, a large amount of precipitation with different morphologies, including rod MgZn_2_ Laves phase (*β’*_1_) and plate MgZn_2_ Laves phase (*β’*_2_), was observed. It has been demonstrated that the main strengthening phase in the current Mg alloy was the *β’*_1_ phase [18]. However, the very different diameter rod from ~12 nm to ~75 nm of the *β’*_1_ phase may be one of the negative influences on the variation of mechanical strength. In addition, the coarsening of precipitated phases was observed after ageing, which may increase the tendency of stress concentration on the grain boundaries and phase boundaries. This could possibly increase the probability of initiation and propagation of crack and flaws [19,20] and result in a relatively variable failure strength.

**Figure 4:**
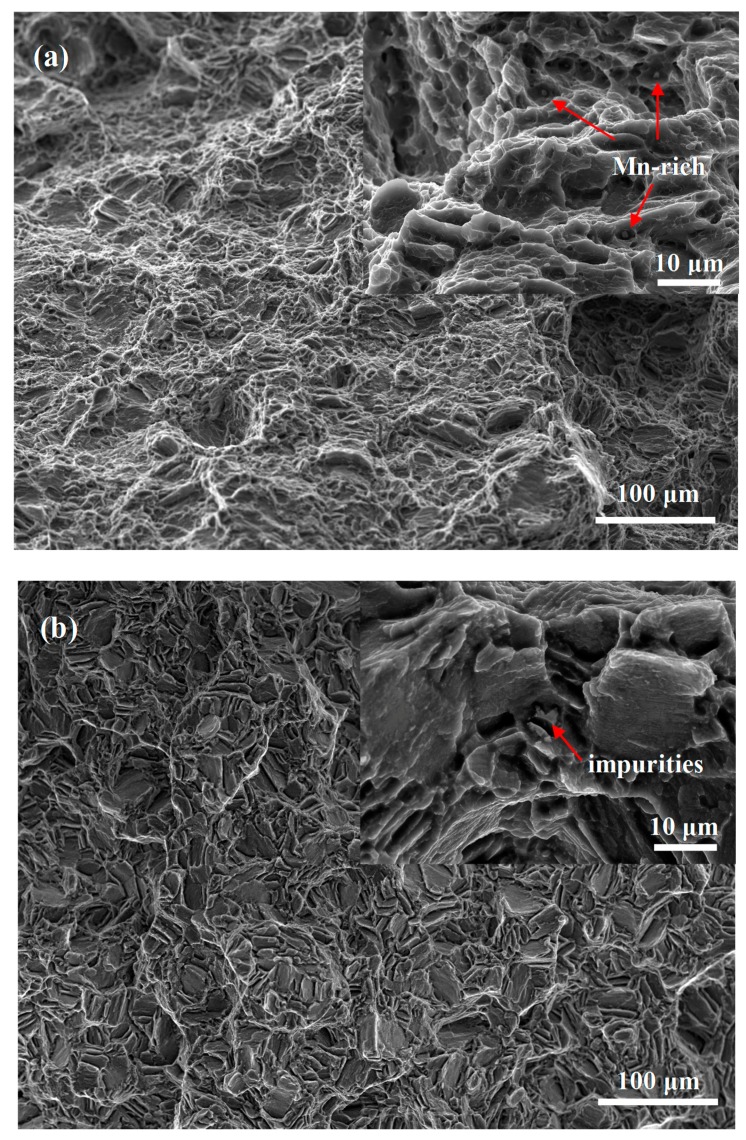
The fracture morphologies of the ZM61 magnesium alloys: (**a**) extruded and (**b**) aged.

These flaws could be further interpreted by the fracture morphologies of the extruded (a) and aged (b) magnesium alloys as shown in Figure 4, which displayed ductile tearing fracture characteristics and typical cleavage fracture features, respectively. The embedded Mn-rich particles in the dimples appeared on the fracture surface as shown in the inset, implying that the extruded alloy did not have any macro-inclusions. In contrast, EDS analysis of the inclusion-initiated fracture in the aged alloy with the lowest *σ_f_* revealed some impurities, such as Fe and Si. It was noted that such defects were usually located at or near the specimen surface, which likely yielded a highly severe stress concentration. These macro-inclusions were also responsible for the relatively large variation of the *σ_f_* for aged Mg alloys. These findings are consistent with the notion that magnesium alloys are capable of large ductility which reduces their flaw sensitivity, as indicated by the strain-to-failure ε (%) that decreased from ~20% to ~10% for the extruded and aged magnesium alloys, respectively.

**Figure 5:**
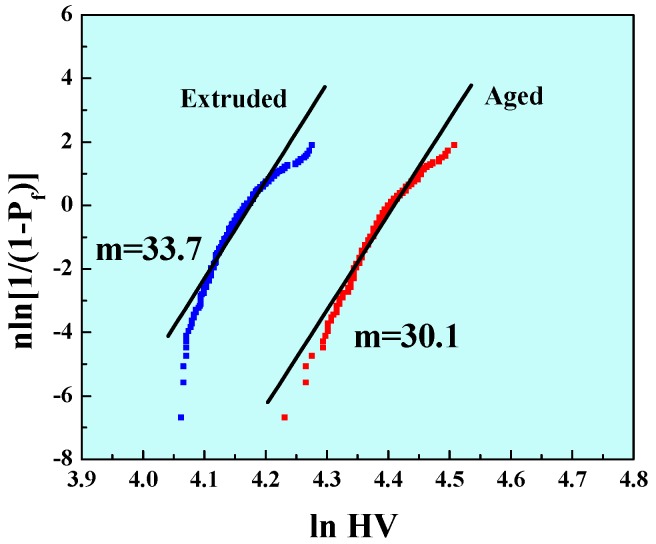
Weibull plot of the microhardness of the extruded and aged ZM61 magnesium alloys.

Furthermore, the varieties of mechanical properties for magnesium alloys may be involved in the solution treatment and artificial aging treatment. These flaws in magnesium alloys were generally micron sized. Therefore, the microhardness could be further explained by the degree of flaws in magnesium alloys under different processing conditions. In order to ensure the reliability of the statistical results, we measured twenty indentations on each sample in well-defined regular arrays, resulting in a total of four hundred raw data points for each alloy and fully reflecting the flaw distribution in extruded and aged magnesium alloys. The experimental data could also be reasonably described by the Weibull distribution equation as shown in Figure 5. It can be seen that the Weibull modulus *m* of microhardness for the extruded magnesium alloys was 33.7, which was much higher than that of the aged ones. This implied that the micro-flaws in extruded alloys had rather uniform sizes. Regardless of the flaw size, the narrow distribution in the measured microhardness was a result of a narrow distribution of the flaw size, large or small, further explaining why the extruded alloys had such a high uniformity, as indicated by the uniformity of the apparent yield strength, ultimate tensile strength and fracture strength values measured for a large number of extruded magnesium alloys.

## 4. Conclusions

By utilizing Weibull analysis, we found that both the extruded and aged MgZnMn alloys demonstrate strikingly high consistency in terms of tensile strength. Specifically, the extruded magnesium alloys have a very high reliability with a Weibull modulus *m* = 92.6 for fracture strength. Such a high Weibull modulus is comparable to many types of aluminum and steel. Compared to the extruded magnesium alloys, the aged alloys show a higher flaw sensitivity, which originates from the embrittlement effect by the distinct microstructural features whereby interconnected coarse Laves phase often precipitates within the alloy matrix. We therefore believe that these findings have very important implications for the safety and reliability assessment of magnesium alloys for lightweight applications.

## Figures and Tables

**Table 1:** Typical values of the Weibull modulus *m* for some materials.

Material	*m*	Ref.
*Traditional Ceramics*: Brick, Pottery, Chalk	<3	[8]
*Engineered Ceramics*: SiC, Al_2_O_3_, Si_3_N_4_	5–10	[8]
*Metals:*		
Aluminum, Steel	90–100	[8]
Extruded magnesium alloy	92.6	This work
Mg-based glassy alloy	5–41	[6,14]
